# Evaluating Formulation-Dependent Chemical Variation and Comparability of Maziren-Wan Preparations via Multi-Component LC–MS/MS Profiling

**DOI:** 10.3390/ph19040577

**Published:** 2026-04-03

**Authors:** Chang-Seob Seo

**Affiliations:** KM Science Research Division, Korea Institute of Oriental Medicine, Daejeon 34054, Republic of Korea; csseo0914@kiom.re.kr; Tel.: +82-42-868-9361

**Keywords:** formulation-dependent variability, chemical consistency, Maziren-Wan, LC–MS/MS, quality marker

## Abstract

**Background/Objectives**: Maziren-Wan (MZRW) is a traditional herbal prescription that has been used for the treatment of chronic constipation and is currently available in the form of granules or decoctions. Given its multi-component nature and various dosage forms, evaluating the chemical consistency of MZRW preparations is important for pharmaceutical quality assessment. The aim of the present study was to compare formulation-dependent chemical profiles of different MZRW preparations using a multi-component analytical approach. **Methods**: An excipient-free reference extract and two commercially available MZRW extract granule products were analyzed using a validated liquid chromatography–tandem mass spectrometry (LC–MS/MS) method operating in multiple reaction monitoring mode. Thirty marker compounds derived from the constituent herbs were simultaneously quantified, and their levels were statistically compared among the preparations. **Results**: Quantitative analysis revealed formulation-dependent variation in the abundance of several marker compounds. Amygdalin and magnoloside A exhibited markedly higher levels in the excipient-free reference extract than in the commercial granule products, whereas sennoside A showed relatively consistent levels across the preparations. **Conclusions**: The results indicate that MZRW preparations sharing an identical herbal composition can exhibit formulation-dependent differences in chemical profiles. Comparative evaluation based on multiple marker compounds may provide useful information for assessing chemical consistency and supporting quality assessment of MZRW preparations formulated under different conditions.

## 1. Introduction

Chronic constipation is a common gastrointestinal disorder that often requires long-term management and is associated with a considerable burden on daily life [1,2]. Although various pharmacological agents, including osmotic laxatives and prokinetic drugs, are widely used, a notable proportion of patients report limited symptom improvement or dissatisfaction with conventional treatments [1,2]. As a result, complementary and traditional medicines continue to be utilized as alternative or adjunctive options, particularly in chronic conditions requiring prolonged administration [2]. In such settings, the pharmaceutical quality and chemical consistency of the administered preparation are important factors influencing reproducibility and reliability.

Maziren-Wan (MZRW; Majain-Hwan in Korean medicine and Mashinin-Gan in Japanese Kampo medicine) is a classical multi-herbal prescription traditionally used for the management of chronic constipation [3,4]. The formulation consists of six medicinal herbs: Cannabis Semen, Paeoniae Radix, Ponciri Fructus Immaturus, Rhei Radix et Rhizoma, Armeniacae Semen, and Magnoliae Cortex [3]. Previous clinical and experimental studies have suggested potential improvements in bowel movement frequency and related symptoms following MZRW administration [3,4]. Given its widespread clinical use across different medical systems and its suitability for long-term intake, maintaining pharmaceutical consistency among MZRW preparations is a relevant consideration.

In contrast to conventional single-compound pharmaceuticals, multi-herbal prescriptions like MZRW comprise an intricate matrix of chemically diverse metabolites [5]. The chemical stability of these profiles is often susceptible to fluctuations driven by extraction protocols, dosage form design, the addition of excipients, and the inherent heterogeneity of raw herbal materials [5,6]. A critical divergence often occurs during the transition from a standardized, excipient-free reference extract to a finalized commercial product, as industrial processing introduces unique variables. These formulation-specific factors can alter the solubility or relative concentration of key analytes, leading to detectable chemical shifts between different preparations [5,6]. Consequently, merely documenting the herbal composition is inadequate for ensuring pharmaceutical equivalence. Therefore, the strategic selection and quantification of multiple marker compounds have emerged as a robust methodology for the quality control of complex herbal systems [5,6,7].

Targeted multi-component analysis using liquid chromatography–tandem mass spectrometry (LC–MS/MS) operated in multiple reaction monitoring (MRM) mode is widely applied for the sensitive and selective quantification of marker compounds in complex herbal preparations [8,9]. Validated LC–MS/MS methods have been successfully employed for the quality evaluation of various multi-herbal prescriptions [8,9]. However, formulation-dependent chemical differences between excipient-free reference extracts and commercially available MZRW products have not been systematically examined. Such comparability assessments are important for ensuring lot-to-lot consistency and supporting pharmaceutical standardization of herbal medicinal products. Therefore, the present study focused on the comparative evaluation of chemical profiles among different MZRW preparations formulated under distinct conditions using a validated multi-component LC–MS/MS approach. By examining formulation-dependent variation in multiple marker constituents, this study aims to provide analytical evidence relevant to pharmaceutical quality assessment of MZRW preparations across different dosage forms.

## 2. Results

### 2.1. Multi-Component Quantitative Profiling of MZRW Preparations

Multi-component quantitative analysis was performed to characterize the chemical profiles of MZRW preparations. An excipient-free reference extract (Sample 1) and commercially available MZRW extract granule products (Samples 2 and 3) formulated under different conditions were analyzed using the validated LC–MS/MS method. Thirty marker compounds originating from the constituent herbs were simultaneously monitored and quantified. The selected marker compounds were chosen to ensure comprehensive representation of all six constituent herbs of MZRW while covering major chemical classes, including lignans, flavonoids, phenolic acids, anthraquinones, coumarins, and glycosides. In addition, these compounds were selected based on their reported relevance as characteristic or bioactive constituents and their frequent use in the quality evaluation of herbal medicinal products. Most of the selected marker compounds were detected in the analyzed preparations, while several constituents showed preparation-dependent detection patterns. The quantitative results for individual marker compounds are summarized in Table 1, which provides an overview of the chemical composition of each MZRW preparation. Representative total ion chromatograms obtained in positive and negative ion modes for the mixed reference standard solution and MZRW samples are shown in Figures S1 and S2.

### 2.2. Comparison of Marker Compound Levels Among Different Preparations

**Figure 1:**
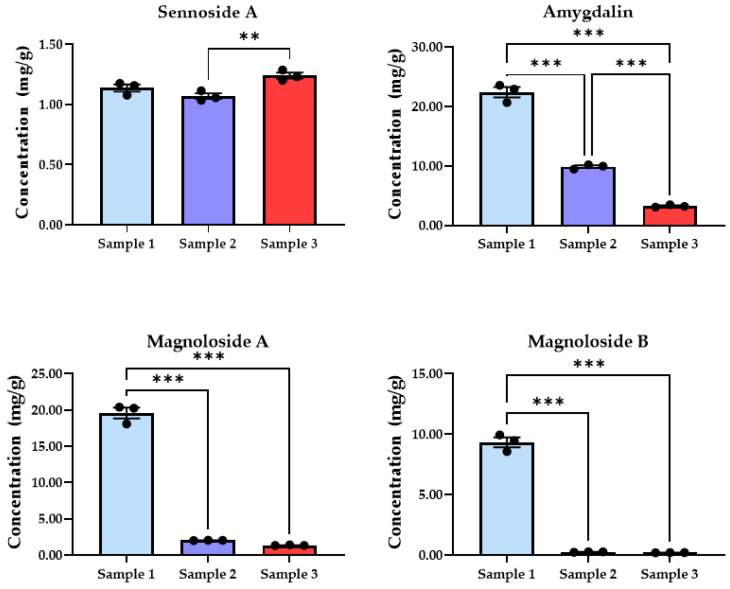

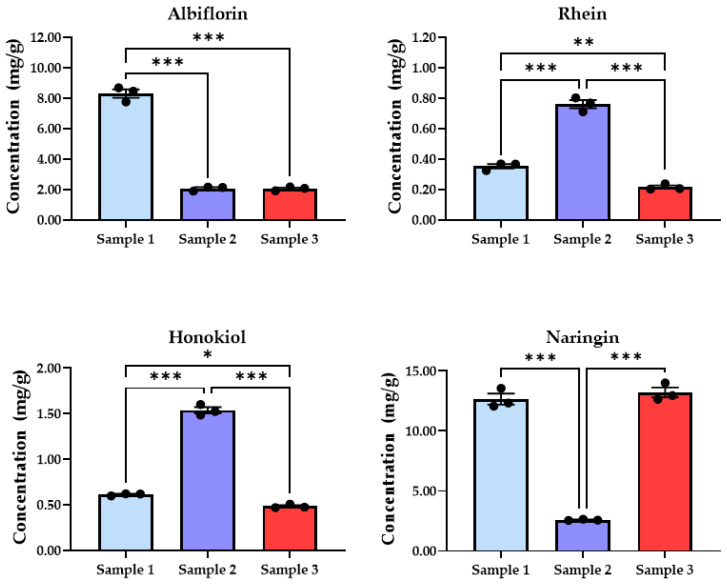
Quantitative comparison of representative marker compounds in MZRW preparations. Eight representative markers were selected to illustrate formulation-dependent chemical variation among the preparations, including sennoside A, amygdalin, magnoloside A, magnoloside B, albiflorin, rhein, honokiol, and naringin. Data are presented as mean ± SD (n = 3). Statistical significance was evaluated using one-way ANOVA followed by Tukey’s multiple comparison test. Asterisks indicate significant pairwise differences between samples (* *p* < 0.05, ** *p* < 0.01, *** *p* < 0.001).

Quantitative comparison indicated differences in the levels of multiple marker compounds among the analyzed MZRW preparations (Table 1). Eight representative marker compounds were selected to illustrate formulation-dependent chemical differences among the preparations (Figure 1). These markers were selected based on their detectability, representation of different herbal sources, and their ability to reflect formulation-dependent variation among the preparations. Among these markers, sennoside A (SNA) exhibited relatively consistent levels across the samples, although a statistically significant difference was observed between Sample 2 and Sample 3 (*p* < 0.01). In contrast, several compounds, including amygdalin (AMY), magnoloside A (MGA), magnoloside B (MGB), and albiflorin (ALB), were detected at higher levels in the excipient-free reference extract (Sample 1) compared with the commercial granule products (Samples 2 and 3) (*p* < 0.001). For instance, the concentration of magnoloside B (MGB) in Sample 1 (9.32 mg/g) was substantially higher than those in Sample 2 (0.25 mg/g) and Sample 3 (0.20 mg/g). A similar trend was observed for magnoloside A (MGA) and albiflorin (ALB), which also reached their highest concentrations in Sample 1. Conversely, some compounds showed relatively higher levels in the commercial products. In particular, anthraquinone-related constituents such as rhein (RHE) and honokiol (HNK) exhibited higher levels in Sample 2 than in the other preparations (*p* < 0.01). Overall, these results indicate compound-dependent differences in the distribution of marker compounds among the MZRW preparations. Detailed quantitative data for all analyzed markers are provided in Table 1, and additional statistical comparisons are presented in the Supplementary Materials (Figure S3).

### 2.3. Herb-Specific Distribution of Marker Compounds

Our analysis indicates that marker distribution is highly herb-specific across the MZRW matrix (Table 1). While constituents derived from Magnoliae Cortex and Paeoniae Radix were ubiquitous across all tested samples, their absolute concentrations fluctuated. SNA, derived from Rhei Radix et Rhizoma, was detected at a concentration range of 1.07–1.24 mg/g across all formulations. In contrast, other anthraquinones from the same source, such as RHE and emodin, were more abundant in the commercial granule products than in the excipient-free reference extract. Furthermore, the major compounds derived from Ponciri Fructus Immaturus showed the highest degree of preparation-dependence, with certain compounds (e.g., isoimperatorin, auraptene, and umbelliferone) detected only in specific commercial granule products.

### 2.4. Variability in Chemical Profiles Across Formulations

Comparison of the quantitative profiling data suggested variability in the overall chemical profiles among the analyzed MZRW formulations (Table 1). Although the preparations shared an identical herbal composition, differences were observed in both the number of detected constituents and their relative abundance. Such variability was observed across multiple compound classes and herbal origins. In contrast, certain marker compounds, including SNA, were detected at comparable levels among all preparations analyzed.

### 2.5. Principal Component Analysis (PCA) of MZRW Preparations

**Figure 2:**
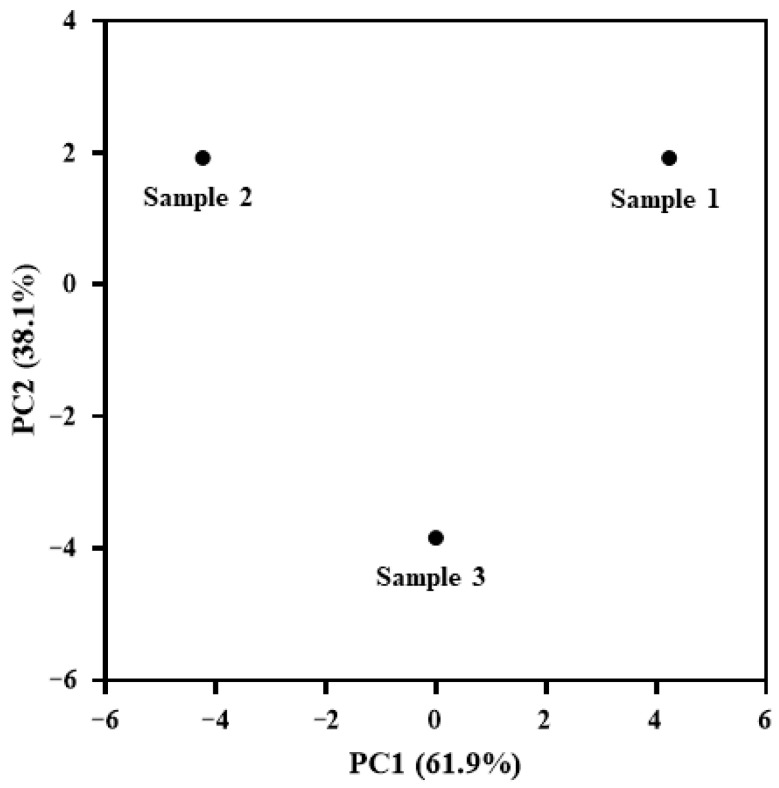
PCA score plot of MZRW preparations based on the quantitative data of 29 marker compounds. PC1 and PC2 explained 61.9% and 38.1% of the total variance, respectively. The separation among Sample 1, Sample 2, and Sample 3 suggests differences in the measured chemical profiling among the preparations.

To further evaluate the overall chemical differences among the MZRW preparations, PCA was performed using the quantitative data of the marker compounds. The PCA score plot showed apparent separation among the three preparations (Figure 2). Apigenin, which was not detected in any of the analyzed samples, was excluded from the PCA dataset. Sample 1 and Sample 2 were separated primarily along PC1, while Sample 3 was separated along PC2, suggesting differences in the measured chemical profiles among the preparations. These results provide a holistic view of the compositional differences and support formulation-dependent variation within the scope of the present dataset.

## 3. Discussion

MZRW preparations sharing an identical herbal composition exhibited noticeable differences in their chemical profiles in the present dataset. Targeted LC–MS/MS quantification demonstrated variation in both the detectability and relative abundance of multiple marker compounds between the excipient-free reference extract and the commercial granule products. Notably, the extent of variation among preparations was compound-dependent. Several lignan- and glycoside-related constituents, including MGA, MGB, and AMY, were detected at substantially higher levels in the excipient-free reference extract than in the commercial granule products. In contrast, certain compounds, such as RHE and HNK, exhibited relatively higher levels in one of the commercial products, suggesting that formulation processes may affect different compound classes in distinct ways.

On the other hand, certain marker compounds showed comparatively stable levels across the analyzed preparations. For example, SNA, an anthraquinone glycoside derived from Rhei Radix et Rhizoma, exhibited relatively consistent concentrations among the samples, although a small difference was observed between Sample 2 and Sample 3. These observations indicate that not all constituents respond equally to formulation or manufacturing processes. Such compound-dependent patterns highlight the importance of evaluating multiple marker compounds when assessing chemical comparability among complex herbal preparations. These findings further support the usefulness of multi-marker approaches for evaluating formulation-dependent chemical variability in herbal medicinal preparations.

The observed differences between the excipient-free reference extract and the commercial granule products may be influenced by multiple factors, including formulation design, processing conditions (e.g., concentration, drying, and granulation), excipient incorporation, and variability in raw materials. While industrial manufacturing processes may affect the solubility, stability, or recovery of specific compounds, these interpretations remain speculative because the manufacturing conditions were not directly examined in the present study. Therefore, the relative contributions of individual factors cannot be definitively distinguished.

From a pharmaceutical quality perspective, the compound- and class-dependent patterns observed in this study indicate that chemical consistency among MZRW preparations cannot be adequately assessed using a single representative marker. Instead, a multi-marker analytical strategy provides a more informative approach for distinguishing formulation-stable markers from formulation-sensitive markers and for characterizing formulation-dependent variability in complex herbal prescriptions. Such an approach may be particularly useful for comparability-oriented quality evaluation of herbal medicinal products that share the same herbal composition but differ in formulation type or manufacturing conditions. These findings also suggest that formulation-dependent variation in chemical composition may lead to variability in pharmacologically relevant constituents, which could potentially influence the consistency of therapeutic effects among MZRW preparations.

The present study has several limitations. The analysis was restricted to a predefined panel of marker compounds selected for targeted LC–MS/MS quantification; therefore, formulation-related changes in minor or uncharacterized constituents were not investigated. In addition, the current experimental design did not allow direct attribution of the observed chemical differences to specific processing variables or excipient–compound interactions. Furthermore, the relationship between chemical variability and pharmacological or clinical outcomes was beyond the scope of the present study. Moreover, due to the limited availability of detailed formulation and manufacturing information for commercial products, it was not possible to clearly distinguish the relative contributions of formulation design, processing conditions, and raw material variability to the observed chemical differences. It should also be noted that method validation was performed using an excipient-free reference extract, and potential matrix effects arising from excipient-containing granule formulations, such as ion suppression/enhancement or differences in extraction efficiency, cannot be entirely excluded.

It should also be noted that the present dataset was based on triplicate analytical measurements for each preparation. Although statistical comparisons were performed to identify significant differences among the preparations, the findings should be interpreted primarily as comparative chemical profiling rather than definitive statistical equivalence assessments. Future studies incorporating a larger number of batches, expanded multi-component profiling, and integration with biological or pharmacological evaluations may provide further insight into formulation-dependent variability and contribute to the development of more robust quality assessment frameworks for herbal medicinal products.

It should be noted that the lower concentrations observed in commercial granule products may be partially attributable to dilution effects caused by excipients, as commercial formulations may contain varying proportions of extract and excipient components depending on the product, which should be considered when interpreting the comparative results. These differences reflect typical formulation and labeling practices and may contribute to variability in the measured marker compound concentrations. In particular, differences in labeling basis (extract amount versus crude drug equivalent) and the presence of excipients may partly account for the observed discrepancies among the products. Therefore, the present comparison should be interpreted as a product-level analytical comparison rather than a direct equivalence assessment.

These findings may have implications for the interpretation of the therapeutic effects of MZRW preparations, as formulation-dependent variation in key constituents could potentially influence their pharmacological activity and contribute to differences in pharmacologically relevant constituents, which may affect the consistency of therapeutic effects. Although the present study did not directly evaluate biological efficacy, the observed differences in chemical profiles support the possibility that formulation-dependent variation may influence the consistency of therapeutic effects. From a quality control perspective, the results highlight the limitations of single-marker-based evaluation and support the use of multi-component analytical approaches for a more comprehensive assessment of herbal medicinal products [5,6,7]. Such strategies may improve the comparability and consistency of formulations produced under different manufacturing conditions.

## 4. Materials and Methods

### 4.1. Chemicals and Reference Standards

Reference standards of the marker compounds analyzed in this study, including lignans, glycosides, flavonoids, phenolic acids, anthraquinones, and coumarins, were obtained from commercial suppliers. The purity of all reference standards was ≥95.0%. Information on chemical structures, compound names, abbreviations, and purity are provided in Figure S4 and Table S1. LC–MS-grade methanol, acetonitrile, and formic acid were purchased from Thermo Fisher Scientific (Waltham, MA, USA). Ultrapure deionized water (resistivity ≥ 18.2 MΩ·cm) was produced using a Milli-Q Integral 15 water purification system (Merck Millipore, Molsheim, France).

### 4.2. Preparation of MZRW Samples

#### 4.2.1. Preparation of the Excipient-Free Reference Extract (Sample 1)

Sample 1 was prepared in accordance with a previously reported extraction procedure [10]. The six herbal medicines constituting MZRW were weighed based on the prescribed formulation ratios (Table S2) to obtain a total of 5.0 kg of mixed raw materials. The herbal mixture was subjected to aqueous extraction with 50 L of deionized water at 100 °C for 2 h using an electric extraction system.

#### 4.2.2. Commercially Available MZRW Extract Granule Products (Samples 2 and 3)

Two commercially available MZRW extract granule products (Samples 2 and 3), manufactured by different manufacturers, were purchased from official distribution channels. According to manufacturer-provided information, both products were formulated as extract granules containing excipients. The granules were pulverized to a homogeneous powder prior to analysis. Limited product information, such as general formulation characteristics, was available from publicly accessible sources; however, detailed data such as batch numbers, extract ratios, recommended daily dose, and quantitative excipient composition were not fully disclosed by the manufacturers. The two products differed in labeling schemes, with one providing individual extract component amounts per dose and excipient information, and the other providing crude drug equivalents together with total extract content.

#### 4.2.3. Sample Solution Preparation for LC–MS/MS Analysis

For LC–MS/MS analysis, sample solutions were prepared using 70% methanol. This solvent was applied consistently to all samples to allow comparative analysis and does not reflect the original manufacturing extraction conditions. Approximately 0.05 g of each sample was accurately weighed and transferred into a 10 mL volumetric flask. The samples were diluted to volume with the extraction solvent, sonicated for 5 min, and vortex-mixed for 1 min. The resulting solutions were filtered through a 0.2 μm hydrophobic polytetrafluoroethylene syringe filter prior to LC–MS/MS analysis.

### 4.3. LC–MS/MS Instrumentation and Analytical Conditions

Quantitative analysis was performed using a Waters Acquity ultra-performance liquid chromatography (UPLC) H-Class system (Waters, Milford, MA, USA) coupled with a Waters TQD triple quadrupole mass spectrometer equipped with an electrospray ionization source. Chromatographic separation was achieved on an Acquity UPLC BEH C18 column (2.1 × 100 mm, 1.7 μm; Waters) maintained at 45 °C, with an injection volume of 2.0 μL. The mobile phase consisted of water containing 0.1% (*v*/*v*) formic acid and acetonitrile, which were delivered using gradient elution. Mass spectrometric analysis was conducted in both positive and negative ion modes using MRM. Detailed chromatographic conditions and parameters for MRM analysis are provided in Tables S3 and S4.

### 4.4. Method Validation

The LC–MS/MS method was validated in accordance with the International Council for Harmonisation Q2 (R1) guideline, with reference to relevant regulatory guidance documents issued by the U.S. Food and Drug Administration and the Korea Ministry of Food and Drug Safety [11,12,13]. Method validation was conducted with respect to linearity, precision, accuracy, repeatability, and stability. Calibration curves were constructed for all analytes, and linearity was confirmed over the corresponding concentration ranges with coefficients of determination (*R*^2^) ≥ 0.99. Limits of detection and quantification were determined at signal-to-noise ratios (S/N) of 3 and 10, respectively. Accuracy was evaluated by recovery tests at three spiked concentration levels (low, medium, and high), while precision was assessed by intra-day (within one day) and inter-day (three consecutive days) repeated analyses at the same concentration levels. Accuracy and precision were evaluated using Sample 1. Analytes not detected in Sample 1 were excluded from the recovery and precision assessment. The stability of marker compounds in prepared sample solutions was evaluated under autosampler storage conditions at 5 °C for three days. All validation parameters were within acceptable ranges, and detailed validation results are provided in Tables S5–S8. Relevant validation parameters, including calibration curves, linear ranges, and sensitivity, are summarized in Table S5, with detailed LC–MS/MS conditions provided in Tables S3 and S4.

### 4.5. Statistical Analysis

Statistical analyses were conducted using GraphPad Prism version 10 (GraphPad Software, La Jolla, CA, USA). Data are presented as mean ± SD (n = 3). The reported values (n = 3) represent repeated analytical injections of the same sample solution and do not reflect sample preparation or batch-to-batch variability. Therefore, the statistical analysis is intended for comparative purposes only and should be interpreted with caution. Differences among the three MZRW preparations were examined using one-way analysis of variance followed by Tukey’s multiple comparison test for post hoc analysis. All tests were two-tailed, and *p* < 0.05 was considered statistically significant. These comparisons should not be interpreted as definitive assessments of product equivalence or batch-level variability. PCA was additionally performed using the quantitative data of the detected marker compounds to explore overall compositional differences among the MZRW preparations.

## 5. Conclusions

Multi-component LC–MS/MS profiling revealed formulation-dependent differences in the chemical profiles of MZRW preparations despite their identical herbal composition. Variations were observed in both the detectability and relative abundance of marker compounds, indicating compound-dependent responses to formulation conditions. These findings suggest that the pharmaceutical quality of MZRW may not be adequately assessed using a single representative marker. Instead, a multi-component perspective that accounts for both formulation-stable and formulation-sensitive markers may provide a more comprehensive approach. This study supports the use of multi-marker analytical strategies for evaluating chemical variability and ensuring the comparability of complex herbal medicinal products.

## Figures and Tables

**Table 1:** Quantitative profiles of marker compounds in excipient-free reference extract and commercially available MZRW extract granule products (n = 3).

Analyte ^1^	Sample 1 ^2^		Sample 2		Sample 3		Source ^5^
	Mean ± SD ^3^ (mg/g)	RSD ^4^ (%)	Mean ± SD (mg/g)	RSD (%)	Mean ± SD (mg/g)	RSD (%)	
MGB	9.32 ± 0.70	7.48	0.25 ± 0.02	8.87	0.20 ± 0.01	2.81	MC
GA	3.32 ± 0.26	7.73	0.65 ± 0.05	8.17	0.67 ± 0.07	9.72	PR
OPAE	0.39 ± 0.001	0.23	0.11 ± 0.01	6.01	0.14 ± 0.01	5.82	PR
SYR	0.20 ± 0.01	7.12	ND	−	ND	−	MC
CGA	0.13 ± 0.01	5.83	0.08 ± 0.01	6.24	0.12 ± 0.003	2.64	CS
AMY	22.41 ± 1.51	6.76	9.90 ± 0.38	3.80	3.26 ± 0.21	6.55	AS
MGA	19.59 ± 1.30	6.61	2.03 ± 0.02	1.10	1.35 ± 0.04	3.34	MC
MGF	0.69 ± 0.06	9.33	0.32 ± 0.02	6.28	0.13 ± 0.004	3.32	MC
ALB	8.30 ± 0.48	5.81	2.07 ± 0.14	6.88	2.06 ± 0.13	6.21	PR
SNA	1.14 ± 0.05	4.56	1.07 ± 0.04	3.80	1.24 ± 0.04	3.45	RRR
4HCA	0.37 ± 0.02	5.94	0.05 ± 0.004	7.76	0.07 ± 0.01	7.24	CS
NRT	1.28 ± 0.09	7.21	0.28 ± 0.02	7.84	0.06 ± 0.002	3.91	PFI
NAG	12.67 ± 0.79	6.27	2.60 ± 0.05	1.87	13.21 ± 0.71	5.39	CS, PFI
PAE	8.20 ± 0.53	6.48	2.71 ± 0.26	9.46	1.85 ± 0.14	7.73	PR
PNC	2.88 ± 0.04	1.25	0.62 ± 0.02	3.32	ND	−	CS, PFI
API	ND ^6^	−	ND	−	ND	−	CS
NAGN	0.01 ± 0.0003	2.58	0.02 ± 0.002	8.99	0.05 ± 0.004	7.71	CS, PFI
BPAE	0.37 ± 0.03	6.98	0.24 ± 0.02	8.98	0.12 ± 0.01	5.37	PR
BER	0.002 ± 0.0002	7.47	ND	−	0.02 ± 0.001	5.02	PFI
AEM	ND	−	0.18 ± 0.02	9.93	ND	−	RRR
RHE	0.35 ± 0.02	6.91	0.76 ± 0.05	6.03	0.22 ± 0.02	8.96	RRR
IMP	0.01 ± 0.001	8.57	0.06 ± 0.001	1.29	ND	−	PFI
EMO	0.03 ± 0.002	6.92	0.17 ± 0.01	4.81	0.03 ± 0.002	7.37	RRR
HNK	0.61 ± 0.01	2.16	1.54 ± 0.06	3.81	0.49 ± 0.02	4.31	MC
IIMP	ND	−	0.01 ± 0.001	8.31	ND	−	PFI
MGL	0.50 ± 0.05	9.07	1.24 ± 0.08	6.60	0.43 ± 0.04	9.21	MC
CHR	ND	−	0.38 ± 0.04	9.54	0.10 ± 0.01	9.93	RRR
PHY	ND	−	0.13 ± 0.01	7.75	ND	−	RRR
AUR	ND	−	0.46 ± 0.04	8.16	ND	−	PFI
UMB	ND	−	0.03 ± 0.002	9.07	ND	−	PFI

^1^ Analyte: MGB, magnoloside B; GA, gallic acid; OPAE, oxypaeoniflorin; SYR, syringin; CGA, chlorogenic acid; AMY, amygdalin; MGA, magnoloside A; MGF, magnoflorin; ALB, albiflorin; SNA, sennoside A; 4HCA, 4-hydroxycinnamic acid; NRT, narirutin; NAG, naringin; PAE, paeoniflorin; PNC, poncirin; API, apigenin; NAGN, naringenin; BPAE, benzoylpaeoniflorin; BER, bergapten; AEM, aloe-emodin; RHE, rhein; IMP, imperatorin; EMO, emodin; HNK, honokiol; IIMP, isoimperatorin; MGL, magnolol; CHR, chrysophanol; PHY, physcion; AUR, auraptene; UMB, umbelliferone. ^2^ Samples: Sample 1, excipient-free reference extract prepared according to the standardized formulation protocol of the Korea Institute of Oriental Medicine; Samples 2 and 3, commercially available MZRW extract granule products purchased through official distribution channels. ^3^ SD, standard deviation. ^4^ RSD, relative standard deviation. ^5^ Source: MC, Magnoliae Cortex; PR, Paeoniae Radix; CS, Cannabis Semen; AS, Armeniacae Semen; RRR, Rhei Radix et Rhizoma; PFI, Ponciri Fructus Immaturus. ^6^ ND, not detected (below the limit of detection).

## Data Availability

The original contributions presented in this study are included in the article/Supplementary Material. Further inquiries can be directed to the corresponding author.

## Supplementary Materials

The following supporting information can be downloaded at: https://www.mdpi.com/article/10.3390/ph19040577/s1, Figure S1: Representative total ion chromatograms (TICs) obtained in positive ion mode for the mixed reference standard solution and MZRW samples. (A) mixed reference standard solution; (B) Sample 1; (C) Sample 2; (D) Sample 3. Peak: 1, SYR; 2, MGF; 3, ALB; 4, 4HCA; 5, NRT; 6, PNC; 7, API; 8, BER; 9, IMP; 10, IIMP; 11, CHR; 12, PHY; 13, AUR; 14, UMB; Figure S2: Representative TICs obtained in negative ion mode for the mixed reference standard solution and MZRW samples. (A) mixed reference standard solution; (B) Sample 1; (C) Sample 2; (D) Sample 3. Peak: 1, MGB; 2, GA; 3, OPAE; 4, CGA; 5, AMY; 6, MGA; 7, SNA; 8, NAG; 9, PAE; 10, NAGN; 11, BPAE; 12, AEM; 13, RHE; 14, EMO; 15, HNK; 16, MGL; Figure S3: Quantitative comparison of additional marker compounds among MZRW preparations; Figure S4: Chemical structures of the reference compounds; Table S1: Chemical information on the 30 reference standard compounds used for LC–MS/MS analysis; Table S2: Composition of the MZRW formulation used in this study; Table S3: LC–MS/MS MRM conditions for simultaneous determination of the 30 marker compounds in MZRW samples; Table S4: Optimized MRM transitions and MS/MS parameters for the quantitative analysis of marker compounds; Table S5: Linear ranges, regression equations, coefficients of determination (*R*^2^), limits of detection (LOD), and limits of quantification (LOQ) for the 30 marker compounds determined by LC–MS/MS; Table S6: Recovery and precision data for the quantitative analysis of marker compounds; Table S7: Evaluation of instrument stability based on retention time and peak area (n = 6); Table S8: Short-term stability of selected marker compounds in sample solutions over three days (%, n = 3).
