# Supplementary material for: Evaluating Formulation-Dependent Chemical Variation and Comparability of Maziren-Wan Preparations via Multi-Component LC–MS/MS Profiling
Source: Pharmaceuticals (Basel). 2026 Apr 3;19(4):577. doi: 10.3390/ph19040577 (PMC13118590; doi:10.3390/ph19040577)
Supplement: Supplementary file 1 [file pharmaceuticals-19-00577-s001.zip › pharmaceuticals-4211881-supplementary.pdf]

Table S1

Chemical information on the 30 reference standard compounds used for LC–MS/MS analysis.

Analyte[1]	Purity (%)	Molecular formula	CAS No.	PubChem CID	Catalog No.	Supplier
MGB	98.4	C35H46O20	116872-05-0	14018784	BP4753	Chengdu Biopurify Phytochemicals Ltd. (Chengdu, China)
GA	100.0	C7H6O5	149-91-7	370	G7384	Merck KGaA (Darmstadt, Germany)
OPAE	98.0	C23H28O12	39011-91-1	21631105	DR10581	Wuhan ChemFaces Biochemical Co., Ltd. (Wuhan, China)
SYR	99.1	C17H24O9	118-34-3	5316860	CFN99281	Wuhan ChemFaces Biochemical Co., Ltd. (Wuhan, China)
CGA	99.7	C16H18O9	327-97-9	1794427	PHL89175	PhytoLab GmbH & Co. KG (Vestenbergsgreuth, Germany)
AMY	99.0	C20H27NO11	29883-15-6	656516	A6005	Merck KGaA (Darmstadt, Germany)
MGA	98.2	C29H36O15	113557-95-2	73189372	CFN99232	Wuhan ChemFaces Biochemical Co., Ltd. (Wuhan, China)
MGF	98.8	C20H24NO4	2141-09-5	73337	CFN98071	Wuhan ChemFaces Biochemical Co., Ltd. (Wuhan, China)
ALB	99.8	C23H28O11	39011-90-0	24868421	016-22201	Fujifilm Wako Pure Chemical Co. (Osaka, Japan)
SNA	99.1	C42H38O20	81-27-6	73111	DR10967	Shanghai Sunny Biotech Co., Ltd. (Shanghai, China)
4HCA	99.2	C9H8O3	501-98-4	637542	082-06521	Fujifilm Wako Pure Chemical Co. (Osaka, Japan)
NRT	99.5	C27H32O14	14259-46-2	442431	BP0985	Chengdu Biopurify Phytochemicals Ltd. (Chengdu, China)
NAG	98.0	C27H32O14	10236-47-2	442428	CFN99555	Wuhan ChemFaces Biochemical Co., Ltd. (Wuhan, China)
PAE	99.4	C23H28O11	23180-57-6	442534	DR10579	Shanghai Sunny Biotech Co., Ltd. (Shanghai, China)
PNC	98.9	C28H34O14	14941-08-3	442456	DR11185	Shanghai Sunny Biotech Co., Ltd. (Shanghai, China)
API	98.1	C15H10O5	520-36-5	5280443	BP0177	Chengdu Biopurify Phytochemicals Ltd. (Chengdu, China)
NAGN	95.0	C15H12O5	67604-48-2	932	N5893	Merck KGaA (Darmstadt, Germany)
BPAE	98.0	C30H32O12	38642-49-8	21631106	DR10582	Shanghai Sunny Biotech Co., Ltd. (Shanghai, China)
BER	98.0	C12H8O4	484-20-8	2355	CFN98766	Wuhan ChemFaces Biochemical Co., Ltd. (Wuhan, China)
AEM	98.0	C15H10O5	481-72-1	10207	A7687	Merck KGaA (Darmstadt, Germany)
RHE	99.9	C15H8O6	478-43-3	10168	275611	Merck KGaA (Darmstadt, Germany)

IMP	98.9	$C_{16}H_{14}O_4$	482-44-0	10212	A0011	Beijing Express Technology Co., Ltd. (Beijing, China)
EMO	98.2	$C_{15}H_{10}O_5$	518-82-1	3220	BP0532	Chengdu Biopurify Phytochemicals Ltd. (Chengdu, China)
HNK	98.0	$C_{18}H_{18}O_2$	35354-74-6	72303	CFN99902	Wuhan ChemFaces Biochemical Co., Ltd. (Wuhan, China)
IIMP	98.0	$C_{16}H_{14}O_4$	482-45-1	68081	BP0785	Chengdu Biopurify Phytochemicals Ltd. (Chengdu, China)
MGL	98.3	$C_{18}H_{18}O_2$	528-43-8	72300	DR10799	Shanghai Sunny Biotech Co., Ltd. (Shanghai, China)
CHR	99.0	$C_{15}H_{10}O_4$	481-74-3	10208	BP0348	Chengdu Biopurify Phytochemicals Ltd. (Chengdu, China)
PHY	98.8	$C_{16}H_{12}O_5$	521-61-9	10639	TB0045	Wuhan ChemNorm Biotech Co., Ltd. (Wuhan, China)
AUR	98.0	$C_{19}H_{22}O_3$	495-02-3	1550607	CFN98787	Wuhan ChemFaces Biochemical Co., Ltd. (Wuhan, China)
UMB	99.8	$C_9H_6O_3$	93-35-6	5281426	DR11382	Shanghai Sunny Biotech Co., Ltd. (Shanghai, China)

[1] MGB, magnoloside B; GA, gallic acid; OPAE, oxypaeoniflorin; SYR, syringin; CGA, chlorogenic acid; AMY, amygdalin; MGA, magnoloside A; MGF, magnoflorin; ALB, albiflorin; SNA, sennoside A; 4HCA, 4-hydroxycinnamic acid; NRT, narirutin; NAG, naringin; PAE, paeoniflorin; PNC, poncirin; API, apigenin; NAGN, naringenin; BPAE, benzoylpaeoniflorin; BER, bergapten; AEM, aloe-emodin; RHE, rhein; IMP, imperatorin; EMO, emodin; HNK, honokiol; IIMP, isoimperatorin; MGL, magnolol; CHR, chrysophanol; PHY, physcion; AUR, auraptene; UMB, umbelliferone.

Table S2

Composition of the MZRW formulation used in this study.

Herbal name	Scientific name	Family	Using part	Origin	Manufacturing No.	Amount (g)	Ratio (%)
Cannabis Semen	*Cannabis sativa* L.	Cannabaceae	Seed	China	K0721201701	1449.28	28.99
Paeoniae Radix	*Paeonia lactiflora* Pall.	Paeoniaceae	Root	Korea	K3472201702	652.17	13.04
Ponciri Fructus Immaturus	*Poncirus trifoliata* L.	Rutaceae	Immature fruit	Korea	K1412201706	652.17	13.04
Magnoliae Cortex	*Magnolia officinalis* Rehder & E.H.Wilson	Magnoliaceae	Bark	China	K0521201701	869.57	17.39
Rhei Radix et Rhizoma	*Rheum tanguticum* Maxim. ex Balf.	Polygonaceae	Root, Rhizome	China	K0101201704	652.17	13.04
Armeniacae Semen	*Prunus armeniaca* L.	Rosaceae	Kernel	China	K0441201704	724.64	14.50
					Total	5000.00	100.00

Table S3

LC–MS/MS MRM conditions for simultaneous determination of the 30 marker compounds in MZRW samples.

**LC conditions**		**MS conditions**	
LC system	Acquity UPLC H-Class	MS system	TQD[1]
Column	Acquity UPLC BEH C_{18} column (2.1 mm × 100 mm, 1.7 μm)	MS software	MassLynx v4.2
Column temp.	45 °C	Ion source[2]	ESI^+ and ESI^-
Sample temp.	5 °C	Acquisition mode	MRM[3]
Injection volume	2.0 μL	Capillary voltage	3.0 kV
Flow rate	0.3 mL/min	Cone gas flow	50 L/h
Mobile phase A	0.1% (v/v) formic acid in deionized water	Desolvation gas flow	500 L/h
Mobile phase B	Acetonitrile	Desolvation temp.	300 °C
		Source temp.	120 °C

	Time (min)	A (%)	B (%)
	0.0	80	20
	0.1	80	20
Gradient	14.0	5	95
	15.0	0	100
	15.1	80	20
	18.0	80	20

[1] TQD, tandem quadrupole detector; [2]ESI, electrospray ionization; [3]MRM, multiple reaction monitoring.

Table S4

Optimized MRM transitions and MS/MS parameters for the quantitative analysis of marker compounds.

Analyte	Ion mode	Exact mass (Da)	Precursor ion (*m/z*)	Product ion (*m/z*)	Cone voltage (V)	Collision energy (eV)
MGB	Negative	786.26	785.5	161.0	30	40
GA	Negative	170.02	169.0	125.0	25	15
OPAE	Negative	496.16	495.4	137.0	40	25
SYR	Positive	372.14	395.2	233.1	30	20
CGA	Negative	354.10	353.2	191.0	20	20
AMY	Negative	457.16	456.4	323.0	32	11
MGA	Negative	624.21	623.4	161.0	30	30
MGF	Positive	342.17	342.4	297.2	30	20
ALB	Positive	480.16	481.4	197.1	20	15
SNA	Negative	862.20	861.5	386.1	45	40
4HCA	Positive	164.05	165.0	147.0	20	10
NRT	Positive	580.18	581.0	273.0	15	15
NAG	Negative	580.28	579.3	271.0	45	30
PAE	Negative	480.16	479.6	120.9	32	25
PNC	Positive	594.19	595.0	287.0	20	20
API	Positive	270.05	271.1	121.0	30	35
NAGN	Negative	272.07	271.1	151.0	30	18
BPAE	Negative	584.19	583.4	121.0	40	25
BER	Positive	216.04	216.9	202.0	30	20
AEM	Negative	270.05	269.1	240.0	40	18
RHE	Negative	284.03	283.0	239.0	20	15
IMP	Positive	270.09	271.2	203.0	25	15
EMO	Negative	270.05	269.1	225.1	40	20
HNK	Negative	266.13	265.3	224.2	45	25
IIMP	Positive	270.09	271.0	203.0	20	10
MGL	Negative	266.13	265.3	247.2	45	20
CHR	Positive	254.06	255.1	181.0	45	20
PHY	Positive	284.07	285.1	211.0	45	25
AUR	Positive	298.16	299.2	163.1	30	15
UMB	Positive	162.03	163.1	107.1	30	20

Table S5.

Linear ranges, regression equations, coefficients of determination (R^2), limits of detection (LOD), and limits of quantification (LOQ) for the 30 marker compounds determined by LC–MS/MS.

Analyte	Linear range (μg/L)	Regression equation[1] $y = ax + b$	R^2	LOD (μg/L)	LOQ (μg/L)
MGB	500 – 8000	$y = 0.41x - 15.33$	0.9954	46.71	140.13
GA	500 – 8000	$y = 0.80x - 33.24$	0.9980	44.87	134.61
OPAE	100 – 1600	$y = 1.1x - 28.73$	0.9969	2.63	7.89
SYR	500 – 8000	$y = 0.16x + 110.90$	0.9998	23.98	71.94
CGA	100 – 1600	$y = 2.80x - 170.04$	0.9952	1.80	5.40
AMY	1000 – 16,000	$y = 0.71x - 370.08$	0.9952	1.13	3.39
MGA	1000 – 16,000	$y = 1.07x + 160.77$	0.9978	12.02	36.07
MGF	50 – 800	$y = 11.91x + 613.98$	0.9971	0.70	2.09
ALB	500 – 8000	$y = 1.88x - 198.71$	0.9960	16.62	49.87
SNA	500 – 8000	$y = 0.87x + 53.48$	0.9990	2.81	8.42
4HCA	250 – 4000	$y = 5.37x + 492.43$	0.9972	10.89	32.68
NRT	100 – 1600	$y = 2.41x - 25.14$	0.9966	7.38	22.15
NAG	1000 – 16,000	$y = 2.07x + 816.12$	0.9962	1.36	4.09
PAE	500 – 8000	$y = 0.19x - 7.14$	0.9974	40.02	120.05
PNC	100 – 1600	$y = 3.61x - 1.36$	0.9981	0.41	1.23
API	250 – 4000	$y = 0.90x + 127.59$	0.9979	10.56	31.68
NAGN	50 – 800	$y = 3.57x - 219.53$	0.9959	0.80	2.40
BPAE	500 – 8000	$y = 0.59x - 74.10$	0.9985	11.09	33.26
BER	10 – 160	$y = 44.08x + 296.14$	0.9965	0.22	0.65
AEM	500 – 8000	$y = 0.74x + 66.25$	0.9965	12.93	38.78
RHE	50 – 800	$y = 5.16x + 306.74$	0.9951	1.46	4.38
IMP	10 – 160	$y = 44.22x + 115.17$	0.9990	0.99	2.98
EMO	100 – 1600	$y = 2.88x + 30.12$	0.9966	2.26	6.77
HNK	250 – 4000	$y = 1.01x + 44.28$	0.9955	4.94	14.81
IIMP	10 – 160	$y = 52.50x + 67.08$	0.9972	0.97	2.91
MGL	100 – 1600	$y = 2.12x + 93.31$	0.9965	1.45	4.35
CHR	250 – 4000	$y = 1.05x - 95.44$	0.9964	11.44	34.33
PHY	100 – 1600	$y = 0.67x - 32.19$	0.9959	3.10	9.29
AUR	500 – 8000	$y = 4.60x + 674.21$	0.9963	3.59	10.77
UMB	25 – 400	$y = 383.79x + 5300.33$	0.9960	0.87	2.61

[1] *y*: peak area of compounds; *x*: concentration (μg/L) of compounds.

Table S6.

Recovery and precision data for the quantitative analysis of marker compounds detected in Sample 1.

Compound	Spiked amount (μg/L)	Recovery (n = 5)			Precision (RSD, %)	
		Mean (%)	SD[1]	RSD (%)[2]	Intra-day (n = 5)	Inter-day (n = 15)
MGB	1000	99.78	2.78	2.78	0.78	2.01
	2000	94.77	3.48	3.67	4.39	4.04
	4000	98.88	6.10	6.17	4.03	4.86
GA	1000	96.50	4.62	4.79	4.32	4.86
	2000	93.54	7.72	8.25	4.31	6.49
	4000	99.86	6.49	6.50	4.19	5.32
OPAE	200	98.99	0.74	0.74	0.72	0.85
	400	101.12	2.57	2.54	1.37	1.83
	800	101.76	5.90	5.80	3.21	4.27
SYR	1000	103.36	4.22	4.09	4.89	3.91
	2000	96.73	4.48	4.64	3.25	3.55
	4000	105.47	5.81	5.51	6.96	9.02
CGA	200	98.83	2.37	2.40	1.37	2.48
	400	99.83	2.15	2.15	3.12	2.15
	800	102.22	6.16	6.03	5.51	4.77
AMY	2000	100.85	0.38	0.37	0.90	1.13
	4000	103.12	2.20	2.13	4.13	2.66
	8000	102.42	1.55	1.51	5.15	3.50
MGA	2000	100.03	2.10	2.10	1.39	1.82
	4000	103.39	1.79	1.73	4.37	2.99
	8000	100.65	2.70	2.68	2.62	2.64
MGF	100	101.16	0.77	0.76	4.38	1.94
	200	103.23	1.90	1.84	6.62	3.87
	400	94.83	8.02	8.46	6.95	6.19
ALB	1000	102.83	0.61	0.59	1.16	1.48
	2000	101.55	1.17	1.16	3.90	3.33
	4000	105.44	1.30	1.23	5.22	2.70
SNA	1000	101.46	0.81	0.80	2.23	1.75
	2000	96.65	3.13	3.24	4.82	3.78
	4000	97.74	5.88	6.01	4.92	4.74
4HCA	500	103.06	0.92	0.89	3.96	2.41
	1000	102.11	1.92	1.88	2.81	3.03
	2000	103.21	2.86	2.77	3.43	4.05
NRT	200	98.59	3.38	3.42	2.22	2.39
	400	101.01	3.11	3.08	2.06	2.57
	800	105.30	5.65	5.37	4.29	5.68
NAG	2000	99.71	3.33	3.34	2.71	2.78
	4000	105.21	0.78	0.74	4.46	2.14
	8000	104.04	3.56	3.42	4.99	3.72
PAE	1000	101.02	2.00	1.98	1.50	1.51
	2000	103.60	2.73	2.63	2.89	2.53

	4000	99.50	4.18	4.20	5.68	4.31
PNC	200	101.01	2.13	2.11	1.06	1.35
	400	102.35	0.75	0.73	1.23	1.35
	800	101.92	1.27	1.25	4.00	2.28
NAGN	100	103.03	5.33	5.17	11.80	8.95
	200	98.82	12.90	13.05	10.13	12.84
	400	105.89	6.93	6.54	7.36	6.75
BPAE	1000	99.28	2.16	2.18	5.63	3.29
	2000	105.16	2.51	2.38	9.97	7.46
	4000	107.24	5.62	5.25	5.91	6.19
BER	20	90.74	10.37	11.43	9.73	11.00
	40	101.48	11.80	11.63	12.35	12.99
	80	106.19	9.57	9.01	10.82	9.76
RHE	100	100.28	2.92	2.91	3.59	2.96
	200	92.07	5.18	5.63	6.75	5.53
	400	100.16	8.57	8.56	1.86	5.22
IMP	20	90.92	2.29	2.52	7.24	4.27
	40	101.11	1.92	1.90	6.26	5.33
	80	103.85	6.99	6.74	12.92	10.35
EMO	200	92.55	10.28	11.11	5.87	6.92
	400	96.76	5.69	5.88	8.85	8.89
	800	111.47	3.54	3.18	3.43	4.36
HNK	500	100.67	1.66	1.65	2.07	1.53
	1000	102.01	2.73	2.68	3.99	3.38
	2000	103.21	1.92	1.86	4.88	4.55
MGL	200	100.01	5.07	5.07	2.20	4.41
	400	97.41	3.90	4.00	5.69	3.73
	800	102.56	8.21	8.00	8.20	9.53

Validation was conducted using Sample 1 (excipient-free reference extract); compounds not detected in Sample 1 were not evaluated for recovery and precision.

[1] SD, standard deviation; [2] RSD, relative standard deviation.

Table S7.

Evaluation of instrument stability based on retention time and peak area variations (n = 6).

Analyte	Retention time (min)			Peak area		
	Mean	SD[1]	RSD (%)	Mean	SD	RSD (%)
MGB	0.81	0.01	1.30	1278.73	101.07	7.90
GA	0.99	0.02	1.90	805.44	72.16	8.96
OPAE	0.99	0.01	1.04	266.94	19.93	7.47
SYR	1.00	0.01	0.63	154.08	2.51	1.63
CGA	1.04	0.01	1.05	259.97	24.71	9.51
AMY	1.15	0.03	2.32	4480.76	247.52	5.52
MGA	1.18	0.02	1.36	6164.16	428.40	6.95
MGF	1.29	0.02	1.37	2964.27	158.48	5.35
ALB	1.38	0.01	0.71	4585.75	371.47	8.10
SNA	1.63	0.02	0.93	863.48	26.79	3.10
4HCA	1.71	0.01	0.49	1857.62	135.33	7.28
NRT	1.84	0.01	0.56	973.71	85.12	8.74
NAG	1.98	0.02	1.14	8982.34	736.95	8.20
PAE	2.39	0.03	1.15	4051.29	316.73	7.82
PNC	3.21	0.01	0.23	1512.04	86.15	5.70
API	3.80	0.02	0.61	304.46	16.52	5.42
NAGN	3.82	0.01	0.31	239.49	22.97	9.59
BPAE	3.86	0.02	0.46	501.83	39.32	7.83
BER	4.87	0.01	0.30	470.71	10.53	2.24
AEM	5.34	0.02	0.36	297.96	19.62	6.58
RHE	5.71	0.02	0.37	912.80	59.00	6.46
IMP	7.29	0.01	0.09	562.24	38.30	6.81
EMO	7.40	0.02	0.28	262.26	21.77	8.30
HNK	7.83	0.02	0.24	552.50	12.82	2.32
IIMP	8.11	0.01	0.15	455.47	38.07	8.36
MGL	8.66	0.01	0.12	548.57	43.48	7.93
CHR	8.98	0.03	0.32	140.21	11.43	8.15
PHY	9.75	0.03	0.35	53.83	3.74	6.95
AUR	10.78	0.01	0.12	1674.28	96.71	5.78
UMB	10.78	0.02	0.19	14459.40	792.59	5.48

[1] SD: standard deviation.

Table S8.

Short-term stability of marker compounds in sample solutions over three days (%, n = 3).

Compound	Time (%)			Mean (%)	SD	RSD (%)
	Day 1	Day 2	Day 3			
MGB	100.00	88.21	94.96	94.39	5.92	6.27
GA	100.00	118.73	105.76	108.16	9.60	8.87
OPAE	100.00	113.21	114.17	109.13	7.92	7.26
SYR	100.00	117.59	106.97	108.19	8.86	8.19
CGA	100.00	100.33	88.83	96.39	6.55	6.79
AMY	100.00	106.46	87.65	98.04	9.56	9.75
MGA	100.00	106.99	90.13	99.04	8.47	8.55
MGF	100.00	112.39	97.85	103.41	7.85	7.59
ALB	100.00	110.60	91.43	100.68	9.60	9.54
SNA	100.00	105.52	87.12	97.55	9.44	9.68
4HCA	100.00	104.44	89.79	98.08	7.51	7.66
NRT	100.00	121.27	110.42	110.56	10.63	9.62
NAG	100.00	106.44	88.25	98.23	9.22	9.39
PAE	100.00	103.88	94.21	99.37	4.87	4.90
PNC	100.00	104.64	118.55	107.73	9.66	8.96
API	100.00	99.47	86.50	95.32	7.64	8.02
NAGN	100.00	101.60	86.96	96.19	8.03	8.35
BPAE	100.00	99.69	84.76	94.82	8.71	9.18
BER	100.00	102.45	85.96	96.14	8.90	9.26
AEM	100.00	93.64	95.73	96.46	3.24	3.36
RHE	100.00	105.69	87.22	97.64	9.46	9.69
IMP	100.00	103.67	97.93	100.53	2.91	2.89
EMO	100.00	99.10	83.56	94.22	9.24	9.81
HNK	100.00	115.87	103.06	106.31	8.42	7.92
IIMP	100.00	102.32	86.26	96.19	8.68	9.03
MGL	100.00	115.90	99.12	105.01	9.45	9.00
CHR	100.00	104.53	119.39	107.97	10.14	9.39
PHY	100.00	87.80	87.93	91.91	7.01	7.62
AUR	100.00	102.67	91.43	98.03	5.87	5.99
UMB	100.00	95.96	82.53	92.83	9.15	9.85

Day 1 values were set to 100.00%, and stability was expressed as the relative percentage of the initial concentration.

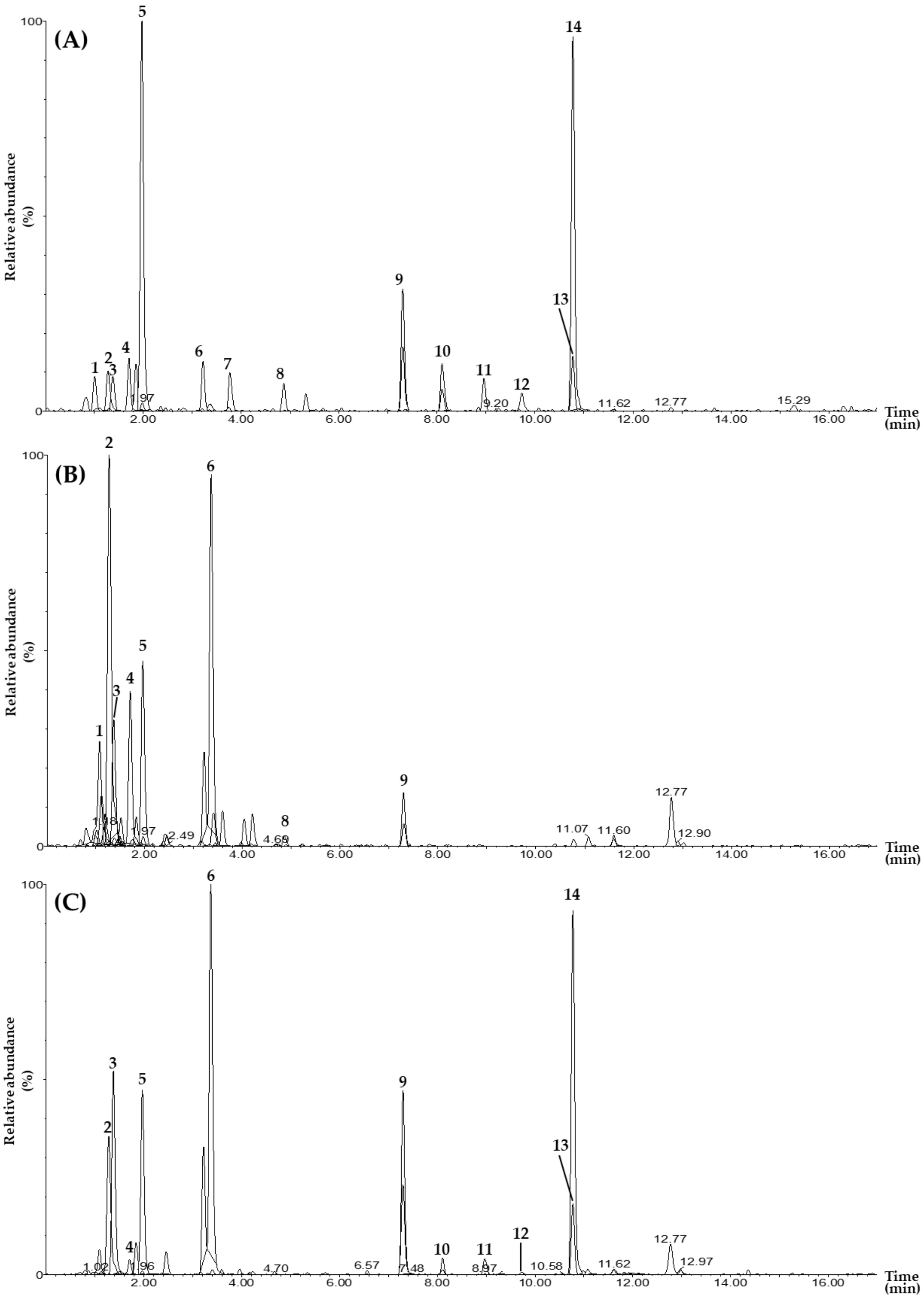
(A)
(B)
(C)
Relative abundance
(%)
Time
(min)
100
0
2.00
4.00
6.00
8.00
10.00
12.00
14.00
16.00
1.97
9.20
11.62
12.77
15.29
2.49
4.69
11.07
11.60
12.90
1.02
1.96
4.70
6.57
7.48
8.97
10.58
12.97

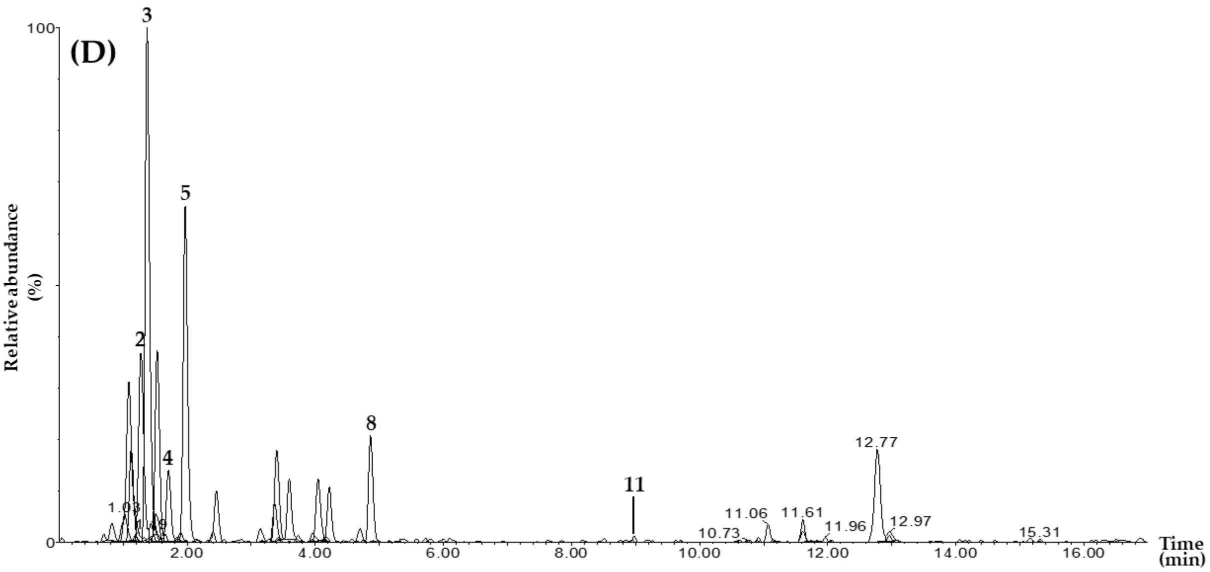

Figure S1. Representative total ion chromatograms (TICs) obtained in positive ion mode for the mixed reference standard solution and MZRW samples. (A) mixed reference standard solution; (B) Sample 1; (C) Sample 2; (D) Sample 3. Peak: 1, SYR; 2, MGF; 3, ALB; 4, 4HCA; 5, NRT; 6, PNC; 7, API; 8, BER; 9, IMP; 10, IIMP; 11, CHR; 12, PHY; 13, AUR; 14, UMB.

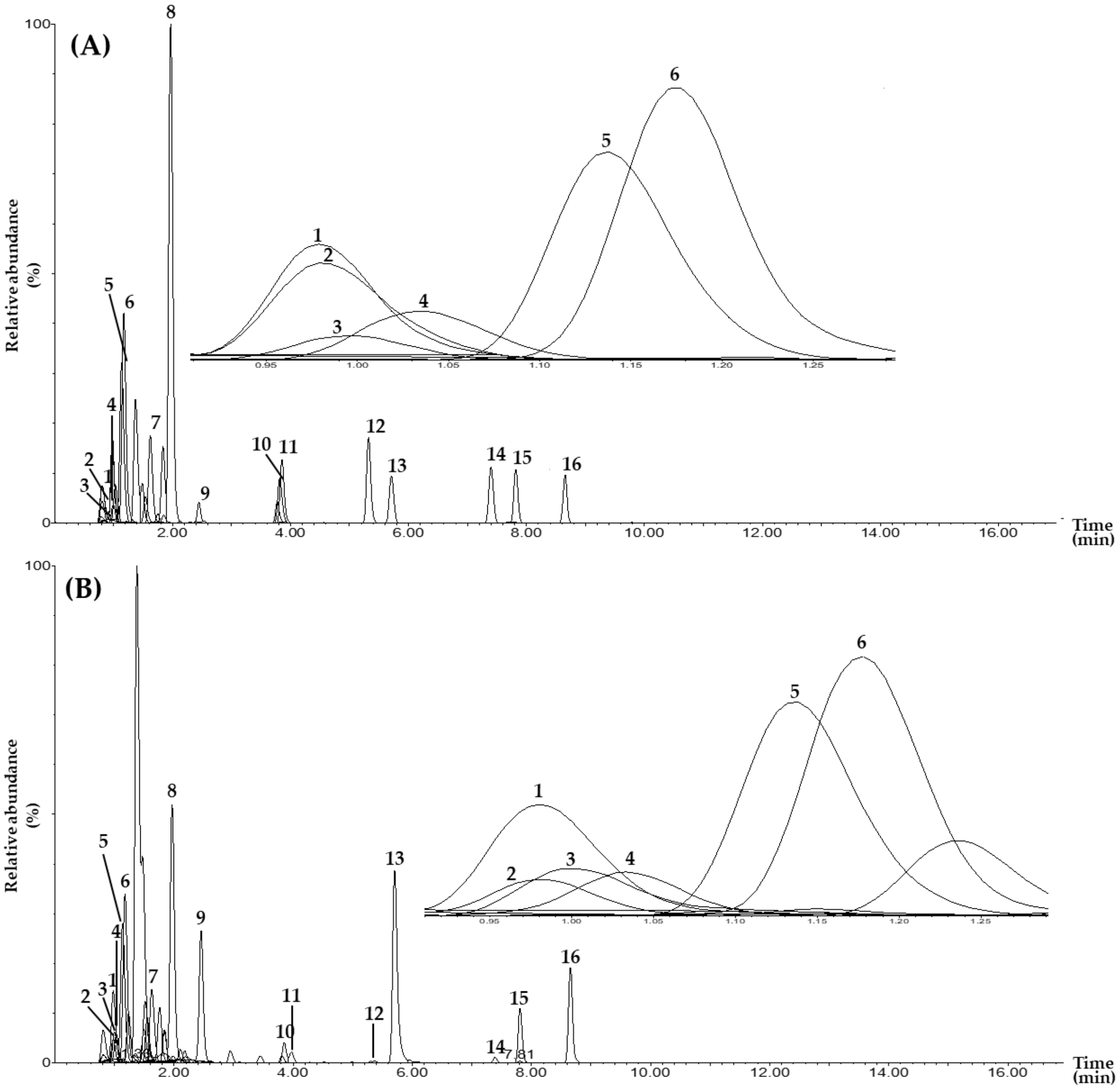
(A)
(B)
Relative abundance (%)
Time (min)
100
0
2.00
4.00
6.00
8.00
10.00
12.00
14.00
16.00
0.95
1.00
1.05
1.10
1.15
1.20
1.25
1
2
3
4
5
6
7
8
9
10
11
12
13
14
15
16

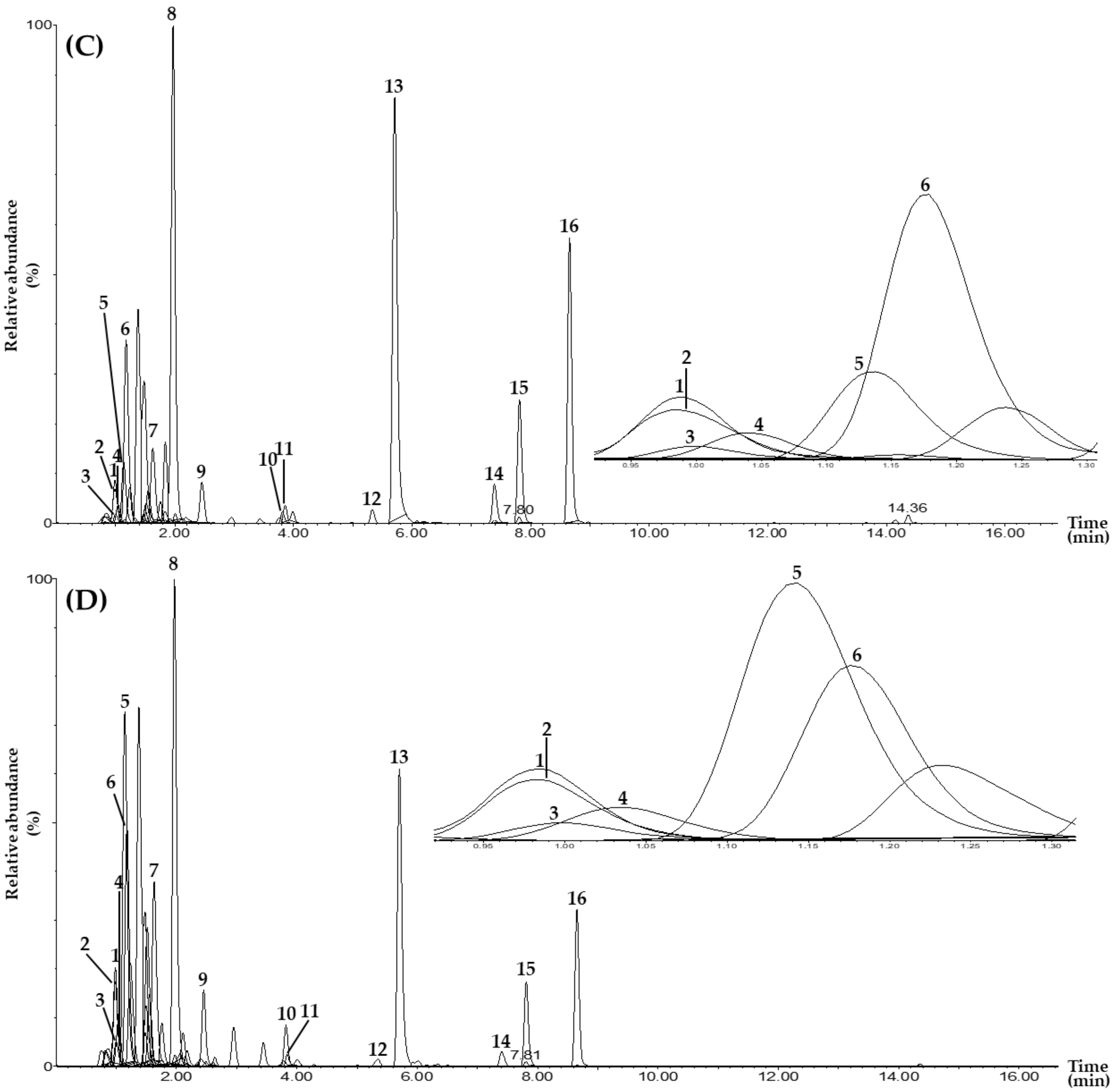

Figure S2. Representative TICs obtained in negative ion mode for the mixed reference standard solution and MZRW samples. (A) mixed reference standard solution; (B) Sample 1; (C) Sample 2; (D) Sample 3. Peak: 1, MGB; 2, GA; 3, OPAE; 4, CGA; 5, AMY; 6, MGA; 7, SNA; 8, NAG; 9, PAE; 10, NAGN; 11, BPAE; 12, AEM; 13, RHE; 14, EMO; 15, HNK; 16, MGL.

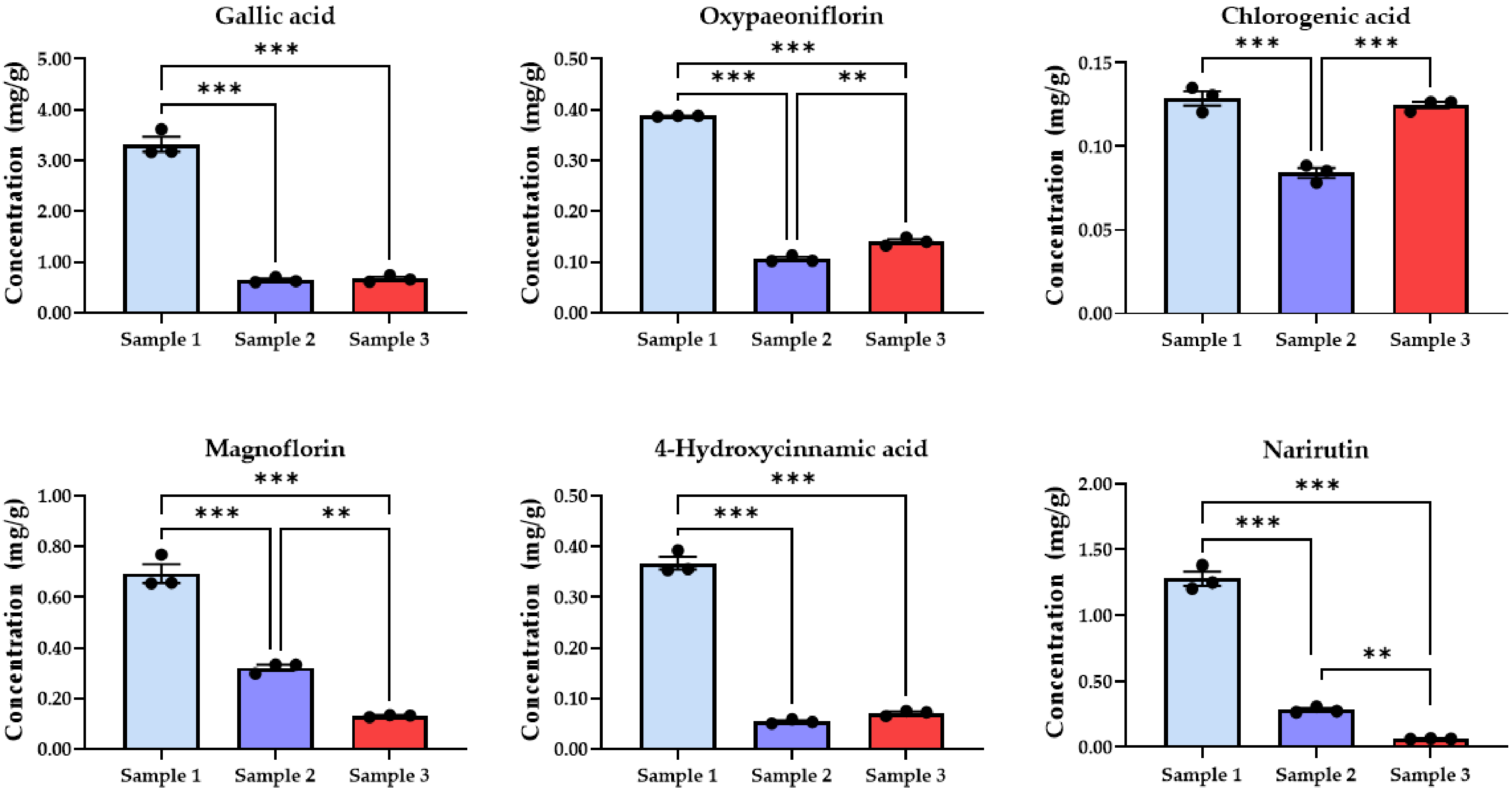

Gallic acid
Oxypaeoniflorin
Chlorogenic acid
Magnoflorin
4-Hydroxycinnamic acid
Narirutin
Concentration (mg/g)
Sample 1
Sample 2
Sample 3
***
**

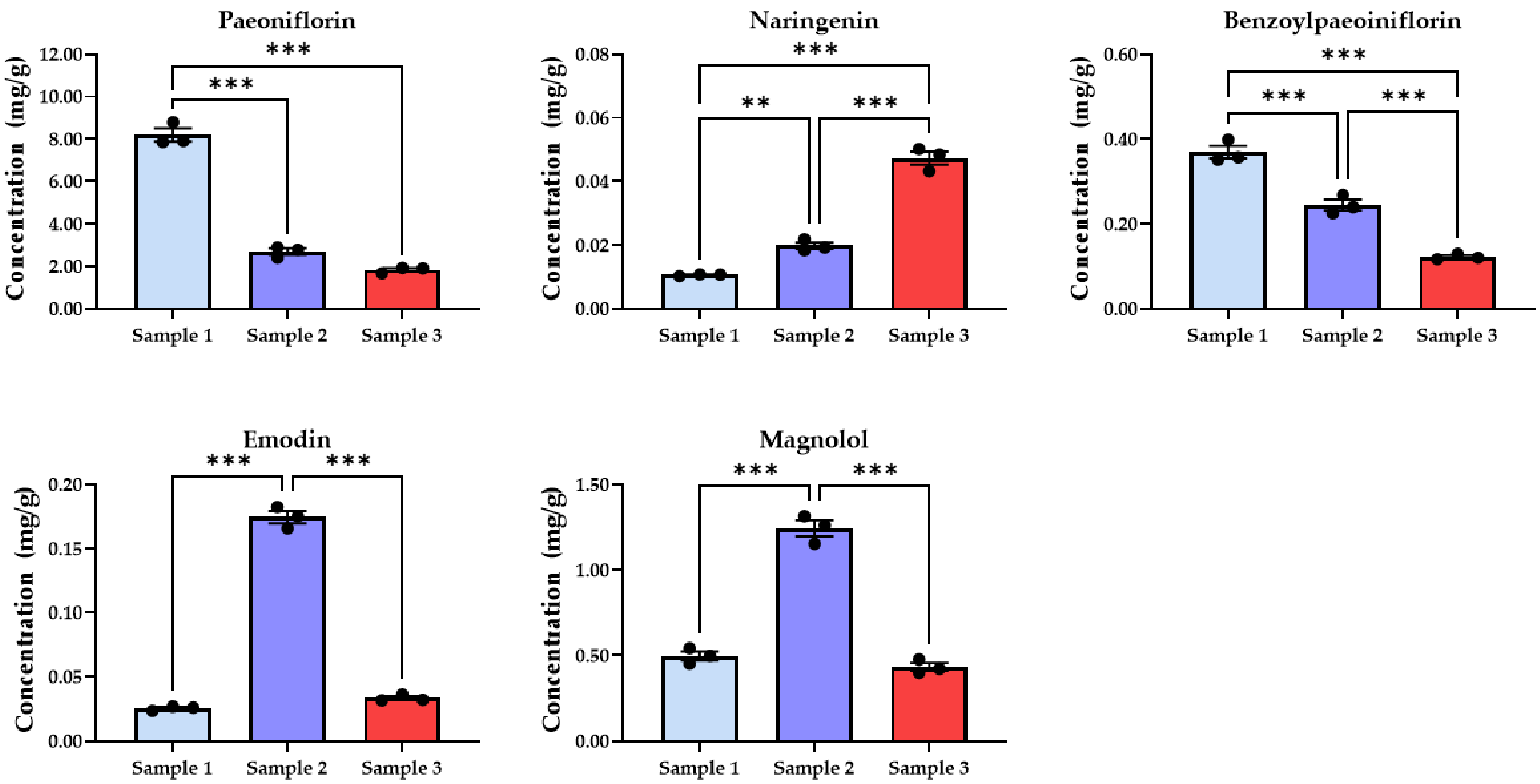

Figure S3. Quantitative comparison of additional marker compounds among MZRW preparations

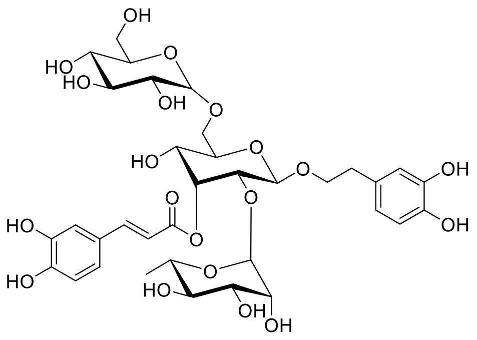

Magnoloside B

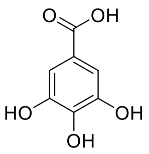

Gallic acid

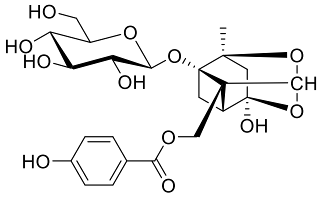

Oxypaeoniflorin

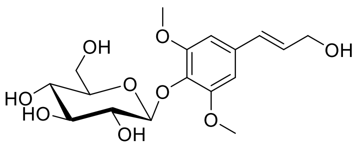

Syringin

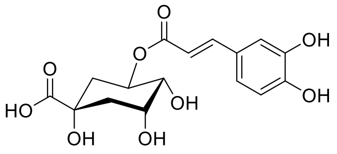

Chlorogenic acid

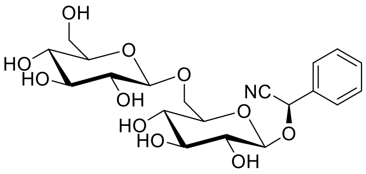

Amygdalin

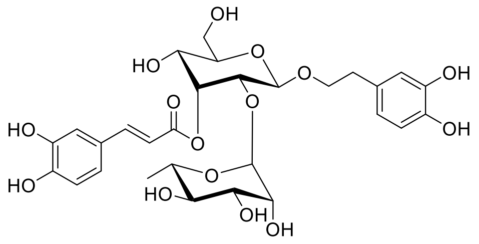

Magnoloside A

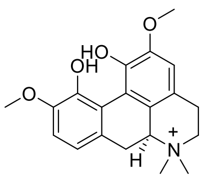

Magnoflorine

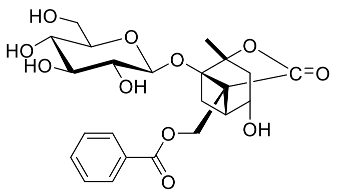

Albiflorin

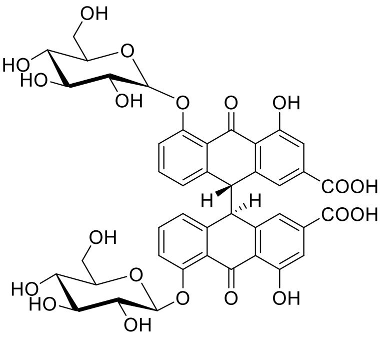

Sennoside A

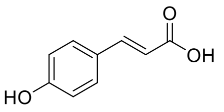

4-Hydroxycinnamic acid

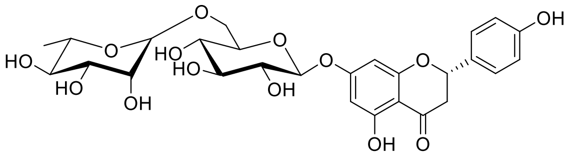

Narirutin

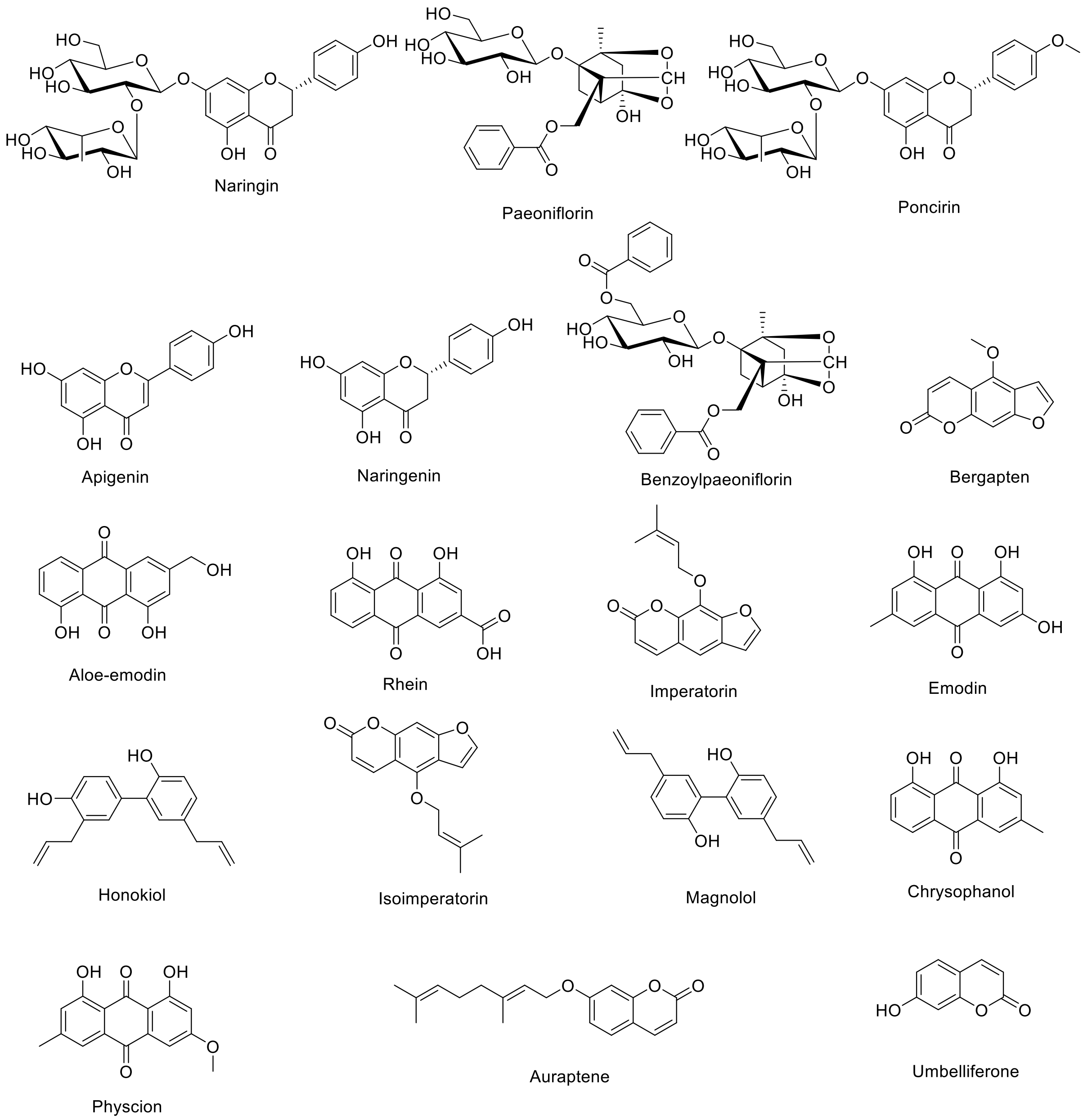

Figure S4. Chemical structures of the reference compounds.
